# Successful renal re-transplantation in the presence of pre-existing anti-DQ5 antibodies when there was zero mismatch at class I human leukocyte antigen A, B, & C: a case report

**DOI:** 10.1186/1752-1947-3-41

**Published:** 2009-01-30

**Authors:** John Hartono, Bhavna Lavingia, Peter Stastny, Martin Senitko, Miguel Vazquez, Juan Arenas, Christopher Lu

**Affiliations:** 1 Division of Nephrology, Department of Internal Medicine, University of Texas Southwestern Medical Center, 5323 Harry Hines Blvd, Dallas, Texas, USA; 2 Division of Transplant Immunology, Department of Internal Medicine, University of Texas Southwestern Medical Center, 5323 Harry Hines Blvd, Dallas, Texas, USA; 3 Department of Internal Medicine, NIH O'Brien Center in Kidney Diseases, University of Texas Southwestern Medical Center, 5323 Harry Hines Blvd, Dallas, Texas, USA; 4 Division of Transplant Surgery, Department of Surgery, University of Texas Southwestern Medical Center, 5323 Harry Hines Blvd, Dallas, Texas, USA; 5 Graduate Program in Immunology, Graduate School of Biomedical Sciences, University of Texas Southwestern Medical Center, 5323 Harry Hines Blvd, Dallas, Texas, USA

## Abstract

**Introduction:**

Hyperacute rejection may be prevented by avoiding the transplantation of kidneys into patients with pre-existing anti-donor Class I human leukocyte antigen antibodies. However, the role of anti-donor-Class II-human leukocyte antigen-DQ antibodies is not established. The question is ever more relevant as more sensitive cross-matching techniques detect many additional antibodies during the final crossmatch. We now report successful renal transplantation of a patient who had pre-existing antibodies against his donor's human leukocyte antigen-DQ5.

**Case presentation:**

Our patient, a Caucasian man, was 34 years of age when he received his first deceased donor renal transplant. After 8 years, his first transplant failed from chronic allograft dysfunction and an earlier bout of Banff 1A cellular rejection. The second deceased donor kidney transplant was initially allocated to the patient due to a 0 out of 6 mismatch. The B cell crossmatch was mildly positive, while the T Cell crossmatch was negative. Subsequent assays showed that the patient had preformed antibodies for human leukocyte antigen DQ5 against his second donor. Despite having preformed antibodies against the donor, the patient continues to have excellent allograft function two years after his second renal transplant.

**Conclusion:**

The presence of pre-existing antibodies against human leukocyte antigen DQ5 does not preclude transplantation. The relevance of having other antibodies against class II human leukocyte antigens prior to transplantation remains to be studied.

## Introduction

Although the risk of hyperacute rejection may be greatly reduced by avoiding the transplantation of kidneys into patients with pre-existing high titers of anti-donor Class I human leukocyte antigen (HLA) antibodies [1] a major recent clinical challenge is understanding the role of low titer antibodies against Class I and Class II HLA molecules. The availability of sensitive modern crossmatch techniques for more HLA antigens makes this challenge cogent [2-4]. Should such pre-existing antibodies prevent transplantation, or should they dictate specific immunosuppressive strategies after transplantation? We now report successful retransplantation in the face of pre-existing anti-donor DQ5 antibodies. Neither plasmapheresis, nor intravenous immunoglobulin was necessary. The contribution of anti-DQ antibodies to rejection would usually be complicated by the presence of mismatches at HLA Class l loci. Our donor-recipient pair is uniquely illustrative because it is matched at Class I HLA A, B, and C, but there were pre-existing donor-anti-recipient HLA Class II DQ antibodies.

The success of this transplantation has potential implications for not only the interpretation of positive crossmatches against DQ, but also for the use of the virtual crossmatch to define "unacceptable" antigens. A PubMed search revealed no reports where a regraft was placed into a patient who had isolated pre-existing anti-donor HLA DQ antibodies.

## Case presentation

In 1991, our patient, a 34-year-old Caucasian male, received his first renal transplant. He did well until 1994 when he had a successfully treated Banff IA rejection associated with non-compliance. After a long course of progressive chronic allograft dysfunction, he returned to dialysis in 1999. His maximal and pre-transplant panel of reactive antibodies was 44% Class I and 80% Class II HLA by screening flow beads (One Lambda, Canoga Park, USA). In addition, analysis of his serum 4 months before his second transplant demonstrated antibodies to HLA-DQ5 that would be present on his second transplant. In July 2005, a kidney from a teenage deceased donor was allocated to our patient because he was thought to have a zero antigen mismatch by conventional serologic assay. The T cell antihuman globulin (AHG) crossmatch and flow T cell crossmatch were negative. The B-cell flow crossmatch was weakly positive, with the molecules of equivalent soluble fluorochrome (MESF) difference of 2308. The threshold for a positive crossmatch was determined by performing a control study with 21 sera from non-sensitized male donors and was fixed at two times the mean plus a standard deviation. Flow cytometry median channel values were converted to MESF using Quantum™ FITC MESF (Low level premix)(QCAL) beads from Bangs Labs (Fishers, IN, USA). The flow crossmatch positive cutoff for our assay was 2254 for the T cell and 2169 for the B cell pronase crossmatch.

**Figure 1:**
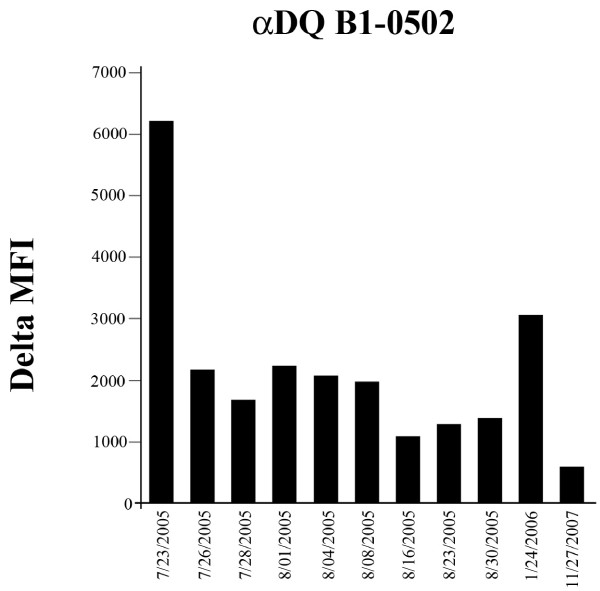
**Serum anti-donor DQ5 antibodies decreased after transplantation**. Antibodies measured using One Lambda beads and a Luminex Analyzer. The transplant was performed on 24^th ^July 2005.

Figure 1 shows the large amounts of serum anti-donor DQ5 antibodies immediately before transplantation and smaller amounts after transplantation. These antibodies were measured using antigen-specific beads from One Lambda and a Luminex analyzer (Austin, TX, USA).

After the transplant, our patient received rabbit anti-human thymocyte globulin, prednisolone, mycophenolic acid and tacrolimus. He was not treated with plasmapheresis or intravenous immunoglobulin. He had excellent initial function and was discharged with a serum creatinine of 1.3 mg/dl. He had no episodes of rejection. His latest serum creatinine 2 years after his second transplant was 1.0 mg/dl.

Table 1 shows the HLA typing for the recipient, as well as for the first and second donor kidneys. The second donor and the recipient were a zero mismatch at HLA Class I A, B and C; they had two allele mismatches at DR and DQ. Note that the first and second transplants shared the Class II HLA DQ 7 antigens.

**Table 1:** Recipient/Donor HLA Typing

		A	B	Bw4/Bw6	Cw	DR/DRB1	DR52/DRB3	DR53/DRB4	DQ/DQB1
Recipient	UNOS	1,11	35,44	w4, w6	w4, w5	4, 17			2,8
	Intermed SSP	1,11	35,44	w4, w6	w4, w5	03,04	pos	Pos	
	High res					*0301 *0402			*0201 *0302

Donor 1	UNOS	2,26	44,22	w4, w6	w5, w9	2,5			6,7
	Intermed SSP		44,55			15,11			

Donor 2	UNOS	1,11	35,-	w6	4,-	4,103			7,5
	Intermed SSP	1,11	35,-	w6	4,-	04, 0103		Pos	03,05
	High res					*0103 *0407			*0301 *0501

## Discussion

We report a successful retransplantation despite pre-existing anti-donor DQ5 antibodies in a donor recipient pair that were zero mismatches at HLA-A, B and C. Neither plasmapheresis nor intravenous immunoglobulin was necessary. A PubMed search revealed no patients who had received a retransplant despite pre-existing anti-donor DQ antibodies. There were also no reports of how a DQ mismatch affected outcome after a regraft. What data is available reports first transplant recipients, as opposed to regrafts, and de novo, as opposed to pre-existing, antibodies. For example, after adjustment for race, age, cold ischemia time, body mass index, immunosuppression, diabetes mellitus and HLA-A, B, and DR match, a study of 12050 first deceased donor transplants found no significant difference between DQ mismatched versus matched kidneys [5]. Other examples are reports on de novo antibodies that appear after transplantation, as opposed to pre-existing, anti-DQ antibodies. Over the short term, de novo antibodies may not cause allograft dysfunction [6]. However, over the long term, de novo anti-DQ antibodies may be associated with chronic allograft glomerulopathy [7]. Surveillance biopsies may detect earlier changes in the allograft, but there are no consensus guidelines for the treatment of chronic allograft dysfunction.

In our recipient, the anti-DQ5 antibodies, found before the second transplant (Figure 1), may have appeared because he was responding to the DQ6 on the retained first transplant. These antibodies may cross-react with DQ5 because DQ5 and 6 are splits of DQ1 and are structurally similar. The anti-donor DQ5 antibodies decreased after transplant perhaps because they bound to endothelia on the DQ5-containing second transplant [8]. However, no allograft dysfunction occurred. One possibility is that the anti-DQ5 may have enhanced survival of stressed endothelia (see review [9]). The persistent decrease in serum anti-donor DQ5 antibodies (Figure 1) suggested either that antibody was absorbed by the transplant or that our immunosuppression prevented formation of new antibodies. Suppression of antibody production has previously been reported for rabbit anti-human thymocyte globulin [10]. Mycophenolic acid and tacrolimus have recently been used to successfully treat some types of acute antibody-mediated rejection [11,12].

Our donor-recipient pair illustrates a potential problem in defining "unacceptable" antigens for a "virtual crossmatch". A DQ mismatch was acceptable in the context of a zero mismatch at HLA A, B and C.

## Conclusion

As progressively more sensitive techniques become available for the final crossmatch, more HLA antibodies are being detected, and are being detected at lower levels. The clinical challenge is deciding if the detected antibodies should prevent transplantation, or should change the immunosuppression [2-4]. One such challenge is determining whether pre-existing anti-donor HLA DQ antibodies increase the risk of acute rejection. Our patient may be uniquely illustrative because he was successfully transplanted despite pre-existing anti-donor HLA-DQ antibodies without plasmapheresis and intravenous immunoimmunoglobulin, and because he was matched at Class I HLA A, B, and C.

## Abbreviations

HLA: human leukocyte antigen; AHG: anti human globulin; MESF: molecules of equivalent soluble fluorochrome; PRA: panel of reactive antibodies.

## Consent

Written informed consent was obtained from the patient for publication of this case report and accompanying images. A copy of the written consent is available for review by the Editor-in-Chief of this journal.

## Competing interests

The authors declare that they have no competing interests.

## Authors' contributions

JH and CL wrote the manuscript and gathered the data. PS and BL performed the tissue typing and the anti-HLA analysis, and helped write the manuscript. JA helped write the manuscript. MS, MV, and CL cared for the patient and provided clinical details.
